# Aberrant degree centrality of functional brain networks in subclinical depression and major depressive disorder

**DOI:** 10.3389/fpsyt.2023.1084443

**Published:** 2023-02-16

**Authors:** Lei Yang, Chaoyang Jin, Shouliang Qi, Yueyang Teng, Chen Li, Yudong Yao, Xiuhang Ruan, Xinhua Wei

**Affiliations:** ^1^College of Medicine and Biological Information Engineering, Northeastern University, Shenyang, China; ^2^Key Laboratory of Intelligent Computing in Medical Image, Ministry of Education, Northeastern University, Shenyang, China; ^3^Department of Electrical and Computer Engineering, Stevens Institute of Technology, Hoboken, NJ, United States; ^4^Department of Radiology, Guangzhou First People’s Hospital, School of Medicine, South China University of Technology, Guangzhou, China

**Keywords:** subclinical depression, major depressive disorder, degree centrality, resting-state fMRI, brain network

## Abstract

**Background:**

As one of the most common diseases, major depressive disorder (MDD) has a significant adverse impact on the li of patients. As a mild form of depression, subclinical depression (SD) serves as an indicator of progression to MDD. This study analyzed the degree centrality (DC) for MDD, SD, and healthy control (HC) groups and identified the brain regions with DC alterations.

**Methods:**

The experimental data were composed of resting-state functional magnetic resonance imaging (rs-fMRI) from 40 HCs, 40 MDD subjects, and 34 SD subjects. After conducting a one-way analysis of variance, two-sample *t*-tests were used for further analysis to explore the brain regions with changed DC. Receiver operating characteristic (ROC) curve analysis of single index and composite index features was performed to analyze the distinguishable ability of important brain regions.

**Results:**

For the comparison of MDD vs. HC, increased DC was found in the right superior temporal gyrus (STG) and right inferior parietal lobule (IPL) in the MDD group. For SD vs. HC, the SD group showed a higher DC in the right STG and the right middle temporal gyrus (MTG), and a smaller DC in the left IPL. For MDD vs. SD, increased DC in the right middle frontal gyrus (MFG), right IPL, and left IPL, and decreased DC in the right STG and right MTG was found in the MDD group. With an area under the ROC (AUC) of 0.779, the right STG could differentiate MDD patients from HCs and, with an AUC of 0.704, the right MTG could differentiate MDD patients from SD patients. The three composite indexes had good discriminative ability in each pairwise comparison, with AUCs of 0.803, 0.751, and 0.814 for MDD vs. HC, SD vs. HC, and MDD vs. SD, respectively.

**Conclusion:**

Altered DC in the STG, MTG, IPL, and MFG were identified in depression groups. The DC values of these altered regions and their combinations presented good discriminative ability between HC, SD, and MDD. These findings could help to find effective biomarkers and reveal the potential mechanisms of depression.

## 1. Introduction

People with major depressive disorder (MDD) often feel terrible or sad, and lose enjoyment in their daily lives. MDD can cause suicidal thoughts in patients and put their lives at risk (1, 2). Subclinical depression (SD) is regarded as the precursor of MDD (3–5). Although the symptoms caused by SD are relatively mild, SD remains a serious disease because it can also cause suicidal ideation (4, 6). The differential diagnosis between MDD and SD is difficult because there is a lack of accurate biomarkers; instead, diagnosis continues to rely on doctors with extensive clinical experience (7).

At present, the pathogenesis of MDD and SD is unclear. One study showed altered activation of the medial prefrontal network regions in MDD (8). The brain regions are related to the anterior cingulate cortex and ventromedial and orbitofrontal cortex. Other studies have found that alterations in frontal-subcortical connectivity in the neural circuits regulating emotion perception (9) may explain the emotional and cognitive symptoms in MDD subjects (10). It is not clear whether the brain regions that are altered in response to MDD are also altered in SD. A study in SD patients indicated that the amplitude of low-frequency fluctuation was significantly increased in the right precuneus and left middle frontal gyrus (MFG), but decreased in the left hippocampus and superior frontal gyrus in SD patients compared to the healthy control (HC) group (5). Another study in mice showed that the lateral habenula is an important brain region that causes depressive symptoms (11). Zhu et al. (4) analyzed the functional connectivity of the lateral habenula in the brains of humans with SD and reported abnormal brain connections related to the thalamus and lateral habenula. The symptoms of SD patients are often relatively mild, making them difficult to identify in time in the early stages of depression. SD has a high probability of developing into MDD, which has a great impact on the lives of patients. Effective measures taken to treat SD patients at an early stage can greatly reduce their suffering and save a lot of medical resources. Therefore, it is important to study the differences between MDD and SD and explore the pathogenesis.

Resting-state functional magnetic resonance imaging (rs-fMRI) is a relatively common means of medical check-up often used in studies of depression. Images are obtained by collecting blood oxygen level dependent images with patients in a resting state during an MRI scan (12, 13). Rs-fMRI is one of the most effective modalities used to detect brain abnormalities (14). Rs-fMRI–based approaches, such as the analysis of the amplitude of low-frequency fluctuations (15), have been widely applied in the examination and study of the pathological mechanisms of various neuropsychiatric diseases (16–19), including MDD and SD (20, 21).

Graph theory is another method to investigate the spontaneous neural activity of the brain from the perspective of a network (22, 23). In the brain network, each brain region is defined as a node, while the functional connectivity (FC) between each pair of regions is defined as an edge. The FC is usually determined by calculating the Pearson correlation coefficient between the time series of rs-fMRI signals of two brain regions. The combination of graph theory and neuroscience can help us explore the mechanisms of our brains further (24, 25). Graph theory has established a mathematical framework to simulate pairwise communication between network elements (26). Graph-based network analysis makes it possible to gather more knowledge about the topological properties of brain networks (24, 27, 28).

The degree centrality (DC) index is an important part of graph theory and network analyses. The degree is a node property, and centrality determines the importance of nodes in a network (24, 29, 30). An investigation of DC can reveal the functional connectivity between each brain region and the rest of the brain in the whole brain (24). Moreover, it can calculate the number of direct connections of a given brain region in the network and reflect the FC of a region in the brain network without a prior choice. The larger the DC value is, the more brain regions are connected to the node of interest. DC has been considered the most reliable indicator among several large-scale network indicators (31), and significant changes in DC may indicate abnormal brain regions (30). Therefore, the DC method has been used to research many psychiatric diseases, including MDD (29, 30), schizophrenia (32), and multiple sclerosis (33). However, few studies assessing DC changes in SD and few studies comparing differences in DC between MDD and SD patients exist. In summary, the above factors motivated us to compare the DC values between the three groups of MDD patients, SD patients, and HCs to reveal the neural basis of MDD and SD and find biological markers of depression.

Alterations in functional brain networks have been proven to exist in MDD and SD (34–36). A few studies reported lower small-worldness of resting-state FC networks in patients with MDD in their 30s as well as in patients with late-life depression undergoing pharmacotherapy (36, 37). Small-world networks have smaller path lengths because they have only a few long connecting edges, which are related to the efficiency of information transfer between regions (36, 37). Weaker small-worldness may indicate decreased efficiency of information transfer between different regions (36). In prior research, resting-state FC networks showed lower network segregation in MDD patients (36–38). One study (36) revealed weaker network segregation in MDD patients in functional connectivity networks by analyzing the clustering coefficients (36–38). Network segregation refers to connections within the brain’s networks, and the clustering coefficient shows the extent to which nodes in a network are clustered together. A large clustering coefficient indicates that the neighbor nodes of this node are closely connected. However, few studies have focused on SD, and whether patients with SD have the same situation remains unknown (34, 39, 40). One study (34) reported that regions with abnormal DC values in SD patients include the caudate nucleus (decreased values) and the MFG (increased values).

In this study, we compared DC values in different groups and aimed to find and analyze brain regions with abnormal DC values. This study included preprocessing of MRI data, brain network construction, DC calculation, statistical analysis, a summary of DC alterations, and receiver operating characteristic (ROC) curve comparisons.

## 2. Materials and methods

### 2.1. Participants

The dataset included 40 patients with MDD (11 men, 29 women), 34 with SD (11 men, 23 women), and 40 HCs (21 men, 19 women) collected from the Guangzhou First People’s Hospital of Guangzhou Medical University. The patients in the MDD group were diagnosed by experienced psychiatrists referring to the Structured Clinical Interview guided by the fifth version of the *Diagnostic and Statistical Manual of Mental Disorders* (41). The Hamilton Rating Scale for Depression (HAMD) (42) scores of each patient were used as auxiliary criteria. The patients in the SD group were also diagnosed by experienced psychiatrists; however, the Beck Depression Inventory II (BDI-II) was used for SD diagnostic criteria (43, 44), and clinical interviews confirmed that patients in the SD group did not meet the criteria for MDD. Finally, HC subjects were required to have no history of mental illness or genetic history of familial psychological disorders, and they were matched in terms of sex, age, and years of education with the patient groups. Every subject was right-handed and had no neurological or psychological disorders. In addition, they were not addicted to alcohol or drugs and did not meet other special conditions.

Volunteer recruitment and image data collection were completed under the supervision of the Medical Ethics Committee of the affiliated Guangzhou First People’s Hospital of Guangzhou Medical University. All subjects were fully aware of the whole data-collection process, and all signed informed consent forms and provided permission for their information to be used.

### 2.2. MRI acquisition

All data were collected using an MRI scanner (Siemens, Germany, 3-Tesla). To reduce the impact of head movement on data quality, foam pads were used to hold patients’ heads in place, and headphones were used to reduce noise from the equipment. All patients met the resting-state scanning conditions, i.e., closing their eyes and staying relaxed and awake during imaging. An experienced doctor reviewed the MRI results to make sure there was no structural damage in the brain.

The scan parameters for the rs-fMRI images were as follows: repetition time (TR), 2,500 ms; flip angle (FA), 90°; echo time (TE), 21 ms; dimension of the matrix, 64 × 64; field of view (FOV), 200 mm × 200 mm; voxel size, 3.5 mm × 3.1 mm × 3.1 mm; slices, 42 with no gap; and echo-planar imaging sequence. There were 200 time points (or volumes) of rs-fMRI data for each subject. Parameters of the T1-weighted images were as follows: FA, 7°; TR, 2,530 ms; TE, 2.34 ms; slice thickness, 1.0 mm with no gap; FOV, 256 mm × 224 mm; and magnetization-prepared rapid acquisition gradient-echo sequences.

### 2.3. Overview of the study procedure

**FIGURE 1:**
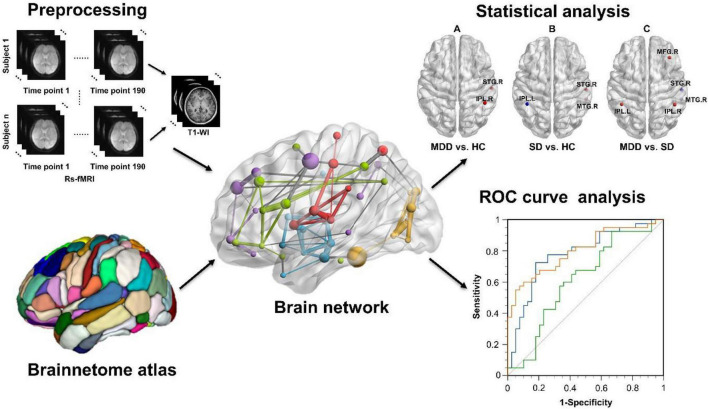
The study design and main procedures included preprocessing, functional brain network construction, statistical analysis of DC values, and ROC curve analysis. DC, degree centrality; ROC, receiver operating characteristic.

The four main procedures of this study are presented in Figure 1 and listed as follows. First, the rs-fMRI and T1-weighted images were preprocessed before further analysis. Second, a brain functional network was constructed for each subject, and the functional connection matrix was obtained. Third, DC calculation and statistical analysis were conducted for the MDD, SD, and HC groups to identify brain regions with significantly different DC values. Finally, ROC curves were analyzed to identify important brain regions.

### 2.4. Preprocessing of rs-fMRI data

The preprocessing step was completed using the Data Processing Assistant for Resting-state fMRI software (45).

1. DICOM images were converted to NIFIT format.
2. To ensure that the MRI equipment was in normal working condition and the subjects had adapted to the scanning process, the first 10 time points were abandoned.
3. The slice-timing step used the middle slice as the reference to eliminate any difference between slices due to acquisition times as much as possible.
4. Slight head movements caused by breathing, heartbeats, or other factors are inevitable. Patients with total scan time <3 min after scrubbing all time points with framewise displacement (FD) >0.2 mm (46) or max head movement with motion >2 mm and rotation >2° were excluded. FD is calculated as the sum of the absolute values of the derivatives of the six realignment parameters (46) and used as the measure of the total absolute movement across the scan.
5. To make the brain images of different subjects more standardized, echo-planar imaging images were registered with T1 images.
6. The brain of each patient was segmented using the “New Segment + DARTEL” method.
7. Covariables (e.g., Friston 24-parameter model and gray matter signal) were removed to reduce the impact on analysis results.
8. Spatial normalization is necessary and it resampled each voxel in the brain to 3 mm × 3 mm × 3 mm.
9. A temporal bandpass filter was applied (0.01–0.08 Hz).
10. Gaussian smoothing was conducted for further noise reduction; the kernel parameter was full width at half-maximum with a size of 6 mm × 6 mm × 6 mm.

### 2.5. Brain network construction

The Graph Theoretical Network Analysis (GRETNA) toolbox (47) was used to complete the functional network constructions based on the data that had been processed. The Human Brainnetome Atlas (48) is one of the most important atlases available to describe the distribution of different brain regions. It divides the brain into 48 brain regions, which are further divided into 246 subregions (nos. 1–246). Using this atlas, the brain can be reconstructed as a network with 246 nodes based on the graph theory method (49). Compared to the Anatomical Automatic Labeling Atlas of 116 brain regions, the Human Brainnetome Atlas was proposed based on connectional architecture and can parcellate the brain on a fine scale. After extracting the time series for any pair of nodes, Pearson correlation coefficients were calculated for these two sequences. This Pearson correlation coefficient can be thought of as an FC between two brain regions. The FC is defined as an edge—an important attribute in graph networks. Finally, brain networks with 246 × 246 matrices for all subjects could be constructed.

### 2.6. DC calculation

There is a lack of a clear standard to select a specific sparsity threshold (47), whereas a principle of determining the threshold is to ensure the integrity and small-worldness (low global efficiency and high local efficiency) of the network. In the current study, a total of 36 binary undirected networks and 36 matrices were obtained using the sparsity threshold of the matrix ranging from 0.05 to 0.4 in increments of 0.01. Here, we empirically chose a threshold of 0.30 for the presentation of the results, as done in previous studies (50, 51). The DC value of a single node is equivalent to the sum of direct connections to other nodes. In an undirected network, the DC value of one node can be measured as:


(1) $$C_{D}\left(N_{i}\right)=\sum_{i=1}^{g}d_{i\,j},\left(i\neq j\right)$$

where *C*_*D*_(*N*_*i*_) is the DC value of the node *N_i_*, *g* represents how many nodes the graph network has, *d*_*ij*_ represents how many edges the network has between *N_i_* and other *g*-1 nodes and *i*≠*j* exclude the connection of *N*_*i*_ with itself.

### 2.7. Statistical analysis

For all subjects, demographic data were analyzed using the relevant statistical module of the Statistical Product and Service Solutions software (SPSS), software program (IBM Corporation, Armonk, NY, USA). When comparing the differences in age and education time, a one-way analysis of variance (ANOVA) was carried out, while the sex differences were obtained by a Chi-squared test. A two-sample *t*-test was performed to analyze the BDI-II scores in the comparison of SD patients and HC subjects.

Statistical analysis was conducted using the GRETNA toolbox. To identify brain regions with significant DC values in the three groups, a one-way ANOVA analysis [Bonferroni corrected, *p* < 0.05, (52)] was performed. Based on the ANOVA results, *post-hoc t*-tests (two-sample *t*-tests) were carried out for the pairwise comparison with a Bonferroni correction (*p* < 0.05). To ensure the quality and accuracy of the statistical analysis for DC, the age, gender, educational level, and head motion were taken as the covariate in the statistical analysis during the ANOVA analysis and the pairwise two-sample *t*-test (MDD vs. HC, SD vs. HC, and MDD vs. SD, respectively). Finally, brain regions with aberrant DC were identified.

### 2.8. ROC curve analysis

For each comparison, the brain regions highlighted by statistical analysis (*p* < 0.05) were extracted for ROC analysis (53) using MedCalc (MedCalc Software, Ostend, Belgium) software. For the comparison of MDD vs. HC, we first analyzed ROC curves for each brain region with abnormal DC values as a single index. Then, all single indexes were combined as a composite index using the logistic regression (LR) method, and the composite index was named LR. Subsequently, the logistic regression (54) process was performed as follows.

In the dichotomous task of distinguishing MDD from HC, we defined *Y* as the category label: *Y* = 0 indicates the HC group and *Y* = 1 corresponds to the MDD group. We defined a total of *N* independent variables *X*_1_, *X*_2_, …, *X*_*N*_. The conditional probability of MDD was *P* = *P*(*Y* = 1|*X*_1_, *X*_2_,…, *X*_*N*_). The logistic regression model is obtained by the following equation:


(2) $$z_{i}=a_{0}+a_{1}\,X_{i\,1}+a_{2}\,X_{i\,2}+\ldots +a_{n}\,X_{i\,N}$$

(3) $$P_{i}=\frac{1}{1+e^{-z_{i}}}$$

where *z_i_* is the intermediate variable parameter (*i* is the number of samples, *i* = 1, 2, …, *M*), *a_0_* is the regression constant, *a_i_* is the regression coefficient of the *j*-th variable (*j* = 1, 2, …, *N*), *X*_*ij*_ is the *i*-th value of the *j*-th variable vector, and *P_i_* is regression prediction probability of disease probability in the *i*-th sample.

Finally, the ROC curves of the composite index were analyzed. ROC curves were compared to analyze distinguishability. Similarly, we compared the ROC curves of every single index with abnormal DC values and the composite index for SD vs. HC and MDD vs. SD using the same method. For the ROC curve analysis, we mainly used the area under the curve (AUC) to analyze the ability to differentiate. In addition, other important measures, such as sensitivity, specificity, and cut-off values, were calculated. Youden’s index represents the ability to distinguish two groups and was used to determine cut-off values.

## 3. Results

### 3.1. Demographic and clinical characteristics

With the standard of scrubbing (scan time <3 min after removing all time points with FD >0.2 mm) and max head movement removal (translation >2 mm and rotation >2°), we ruled out two MDD, eight SD, and seven HC subjects before further data analysis. Table 1 summarizes the demographic and clinical data for all subjects who participated in the data analysis. The statistical analysis of sex, age, and educational level showed that no significant difference (*p* > 0.05) was found. The symptom scores (on the HAMD scale) of the MDD group were significantly different from those of the HC group (*p* < 0.001).

**TABLE 1:** Demographic and clinical characteristics.

Category	HC (*n* = 33)	SD (*n* = 26)	MDD (*n* = 38)	*p*-Value
Gender (female/male)	16/17	16/10	27/11	0.150^a^
Age (years)	19.24 ± 0.94	19.65 ± 1.77	21.13 ± 6.17	0.120^b^
Education (years)	13.18 ± 0.87	13.36 ± 0.92	12.94 ± 2.60	0.492^b^
BDI-II score	1.55 ± 1.44	22.46 ± 7.73	–	<0.001^c^
HAMD score	–	–	21.51 ± 4.58	–

^a^Chi-squared test.

^b^One-way analysis of variance.

^c^Two-sample *t*-test.

### 3.2. Statistical analysis of DC

**FIGURE 2:**
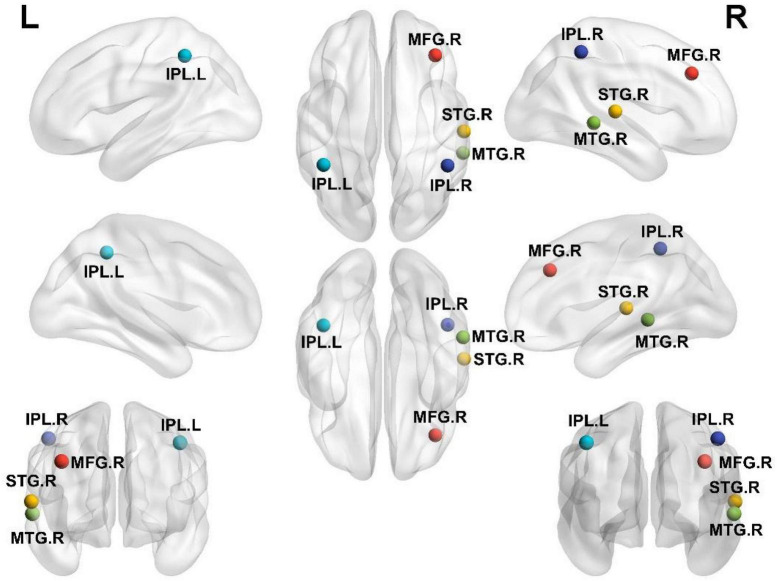
Brain regions with significantly different DC values among the HC, SD, and MDD groups. L, left; R, right; IPL, inferior parietal lobule; MFG, middle frontal gyrus; MTG, middle temporal gyrus; STG, superior temporal gyrus.

According to one-way ANOVA analysis, brain regions with obvious changes in DC included the right MFG, right superior temporal gyrus (STG), right middle temporal gyrus (MTG), and the left and right inferior parietal lobules (IPLs). Figure 2 shows the regions in the brain with altered DC values.

**FIGURE 3:**
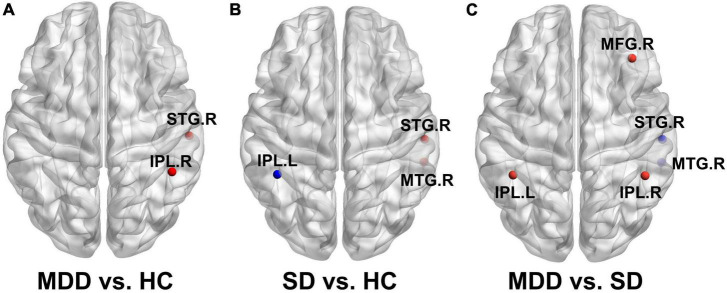
Brain regions with significantly different DC values in inter-group comparisons. **(A)** Brain regions with significant differences in DC between the MDD and HC groups; **(B)** brain regions with significant differences in DC between the SD and HC groups; **(C)** brain regions with significant differences in DC between the MDD and SD groups.

Figure 3A shows DC differences between the MDD and HC groups; notably, the right STG and right IPL possessed increased DC in the MDD group. Higher DC in the right STG and right MTG were found in the SD group compared with the HC group. On the contrary, the left IPL was weaker in the SD group (Figure 3B). Figure 3C shows the regions that differ between MDD and SD groups. Considering the changes in DC in the MDD group, the right MFG, right IPL, and left IPL all possessed a trend toward increased values, while the right STG and right MTG showed decreased DC values, respectively.

**FIGURE 4:**
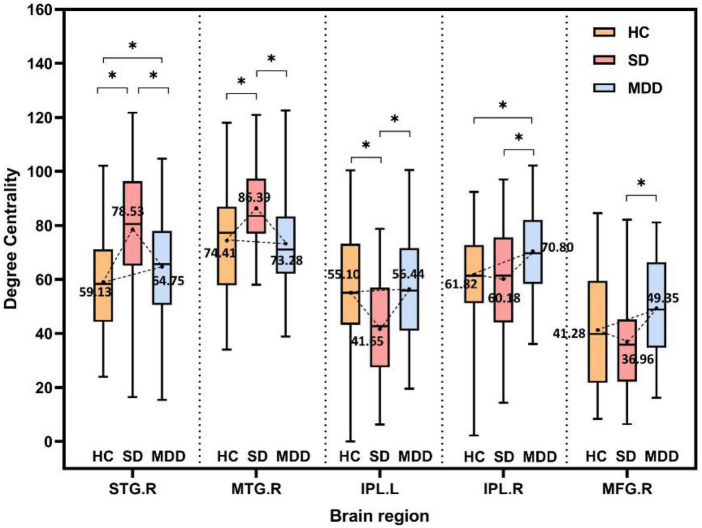
The alteration tendency in DC between HCs, SD, and MDD for five important brain regions. “*” indicates significantly different DC values between the two groups.

According to the comparison results, the alteration tendency of DC in three different stages for each important brain region is summarized in Figure 4. In each comparison, the STG particularly showed significant differences.

### 3.3. Analysis of ROC curves

We compared the ROC curves of brain regions with aberrant DC using the single index and the composite index regressed by the single index, separately. For the aberrant brain regions in each comparison, the AUC value, cut-off point, sensitivity, and specificity of ROC analysis are given in Table 2. AUCs >0.7 indicate a good discriminative ability, and a larger AUC value suggests a greater classification capability.

**TABLE 2:** Analyses of ROC curves for brain regions with altered DC.

Categories	Brain regions	AUC	95% CI	Cut-off point	Sensitivity (%)	Specificity (%)	*p*-Value
MDD vs. HC	STG.R	0.779	0.671–0.865	64.72	72.50	82.05	<0.0001
	IPL.R	0.615	0.499–0.723	53.17	92.50	33.33	0.0752
	LR	0.803	0.698–0.884	–	55.00	94.87	<0.0001
SD vs. HC	STG.R	0.636	0.516–0.745	77.56	41.18	85.00	0.0396
	MTG.R	0.669	0.550–0.774	73.03	82.35	47.50	0.0071
	IPL.L	0.688	0.570–0.791	48.31	70.59	67.50	0.0027
	LR	0.751	0.637–0.844	–	91.18	50.00	<0.0001
MDD vs. SD	MFG.R	0.679	0.560–0.783	41.42	65.00	70.59	0.0050
	STG.R	0.681	0.562–0.785	75.00	75.00	67.65	0.0051
	MTG.R	0.704	0.587–0.805	77.72	60.00	76.47	0.0008
	IPL.L	0.695	0.577–0.797	48.30	65.00	70.59	0.0015
	IPL.R	0.651	0.531–0.758	53.19	92.50	38.24	0.0208
	LR	0.814	0.707–0.895	–	70.00	79.41	<0.0001

**FIGURE 5:**
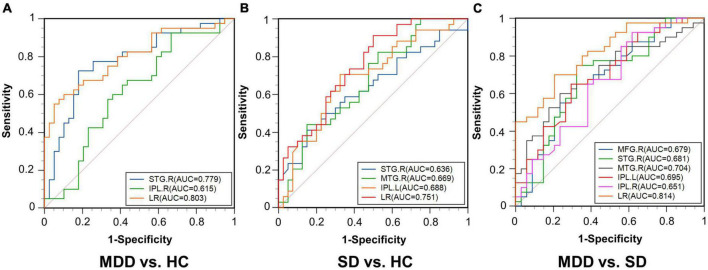
Receiver operating characteristic curves for the three comparisons. **(A)** MDD vs. HC; **(B)** SD vs. HC; **(C)** MDD vs. SD. AUC, area under the curve; STG.R, right superior temporal gyrus; IPL.R, right inferior parietal lobule; LR, logistic regression; MTG.R, right middle temporal gyrus; IPL.L, left inferior parietal lobule; MFG.R, right middle frontal gyrus.

The ROC results of the right STG, right IPL, and the composite index of the right STG and right IPL for MDD vs. HC are shown in Figure 5A. When distinguishing patients with MDD from HCs, the right STG performed well with an AUC of 0.779 [95% confidence interval (CI), 0.671–0.865; *p* < 0.0001]. Conversely, the distinguishing ability of the right IPL was not good, with an AUC of 0.615 (95% CI, 0.499–0.723; *p* = 0.0752), while the AUC of the composite index was 0.803 (95% CI, 0.698–0.884; *p* < 0.0001). Thus, according to the AUC >0.7 rule, the right STG and the composite index showed good discriminative ability.

For the comparison between the SD and HC groups, the ROC results of the right STG, right MTG, and left IPL and the composite index of these regions combined are shown in Figure 5B. When distinguishing patients with SD from HCs, the right STG presented an AUC of 0.636 (95% CI, 0.516–0.745; *p* = 0.0396), the right MTG presented an AUC of 0.669 (95% CI, 0.550–0.774; *p* = 0.0071), and the left IPL presented an AUC of 0.688 (95% CI, 0.570–0.791; *p* = 0.0027), while the AUC of the composite index was 0.751 (95% CI, 0.637–0.844; *p* < 0.0001). The DC value of the composite index had a good discriminative ability.

For the comparison between the MDD and SD patient groups, the ROC results of the right MFG, right STG, right MTG, left IPL, and right IPL and the composite index of these regions combined are shown in Figure 5C. When distinguishing patients with MDD from patients with SD, the AUCs of the right MFG, right STG, right MTG, and left IPL was 0.679 (95% CI, 0.560–0.783; *p* = 0.0050), 0.681 (95% CI, 0.562–0.785; *p* = 0.0051), 0.704 (95% CI, 0.587–0.805; *p* = 0.0008), 0.695 (95% CI, 0.577–0.797; *p* = 0.0015), and 0.651 (95% CI, 0.531–0.758; *p* = 0.0208), respectively. Additionally, the AUC of the composite index was 0.814 (95% CI, 0.707–0.895; *p* < 0.0001). As such, the right MTG and the composite index showed good discriminative ability.

## 4. Discussion

We identified aberrant DC and summarized a tendency for DC alteration by comparing three groups (MDD, SD, and HC) for the first time in the existing study. Notably, the MFG, STG, MTG, and IPL are important areas of the brain that displayed changed DC values. The ROC curve analysis showed that the right STG performs well for the differentiation between MDD and HC groups, and the right MTG could effectively distinguish the MDD and SD groups. All three composite indexes had a good discriminative ability when distinguishing two groups in each pairwise comparison. These findings have implications for the study of depression and will be further discussed and analyzed below.

### 4.1. The STG as an important region in MDD and SD

In each group of comparison experiments, the DC value of the right STG was significantly changed. For both the MDD group and the SD group, the right STG presented an increased DC trend. For the comparison of DC in the MDD and SD groups, the right STG showed a decreased tendency in the MDD group.

As one of the important brain regions, the STG plays a key role in dealing with tasks related to social cognition, emotional processing, and language expression (55, 56). MDD is the main risk factor for suicide. Some studies (57–63) suggest that STG is also associated with depressive symptoms (57) and suicide (58, 59). In a study of depressed patients with suicidal tendencies, the volume of the right STG was reduced (58, 59). In other studies investigating reward-based learning processes, the STG volume was also found to be reduced (60) and the STG was activated (61) in patients with depression. It is reasonable to speculate that the STG’s constant activation may cause volume reduction and trigger depressive symptoms associated with suicide. We speculate that the observed decrease in DC may be related to the reduced volume of the right STG.

In our study, the right STG in the MDD and SD groups presented a higher DC. Zhang et al. (64) observed greater regional homogeneity (ReHo) in the left STG in MDD patients than that in HCs, which also suggests the existence of abnormalities in the STG. ReHo is a reliable measure for reflecting spontaneous neural activity. Under this method, it is assumed that the blood oxygen level dependent signal in a voxel is temporally similar to its neighbors, and Kendall’s coefficient concordance is used to measure the similarity between them. As the prodrome of MDD, SD is likely to develop into MDD (4, 5). We hypothesized that STG changes would be greater in the MDD group because MDD patients had lower DC values than SD patients in the right STG in this analysis.

### 4.2. MTG in MDD and SD

The right MTG in the SD subjects showed higher DC values in the three groups. In our previous studies, the FCs based on the region of interest of the posterior parietal thalamus (PPtha) were investigated because PPtha is an important brain region associated with depression (11, 65). The brain connections between PPtha and the right MTG showed a decreased trend in the MDD group (66). Some studies (58) have indicated that the thalamic-temporal lobe FC was altered in patients with depression. Zhang et al. (64) found that the ReHo of the left MTG in the MDD and SD groups was significantly increased compared with the HC group, and the increasing trend was more obvious in the SD group (64).

In SD patients, the FC, ReHo, and DC all showed decreasing tendencies in the MTG in the MDD group. Therefore, the function of the MTG might be further damaged in more severe stages of depression. As one of the most important areas in the temporal cortex, the MTG is usually associated with the neural response to negative stimuli (64, 67). In addition, the function of the temporal cortex is also related to emotional processing and social cognition (66–68).

As shown in Figure 4, DC values do not necessarily show a strict linear correlation with disease severity. The right STG and right MTG have larger DC values in the SD group than both the HC and MDD groups, while the left IPL has smaller DC values in the SD group than that in the HC group or MDD group. This hypothesis of linear correlation may be too partial or simple in many diseases, considering the complexity of both the disease and brain structures. First, increased or decreased DC values can occur in different impaired brain regions. One previous study (34) showed that SD patients have lower DC values in the right parahippocampal gyrus and left amygdala but higher DC values in the right posterior parietal lobule. Li et al. (69) reported that the DC value is lower in the left triangular part of the inferior frontal gyrus but higher in the left hippocampus in patients with MDD. This discrepancy is not unique to depression-related disorders; patients with diabetic nephropathy and retinopathy have lower DC values in the right inferior temporal gyrus and left subcallosal gyrus regions and higher DC values in the bilateral precuneus (70). In this study, a similar pattern was observed for different indexes in the comparison of the three groups. A higher ReHo was present in the right MTG in the SD group than in either the MDD or HC group, while the ReHo was higher in both the SD and MDD groups than that in the HC group (64). In another study, the right superior frontal gyrus had a lower amplitude of low-frequency fluctuation value in the mild cognitive impairment group than that in the Alzheimer’s disease and HC groups (18). Second, some studies indicate there no significant correlation exists between DC values and disease severity; for example, Gao et al. (34) found that DC values do not correlate with BDI scores in the brain regions with altered DC values in SD subjects. Another study (56) showed that DC values of Parkinson’s disease patients with freezing of the gait did not significantly correlate with the freezing of the gait questionnaire scores for the nine regions with significantly different DC values (*p* > 0.05).

### 4.3. The composite index had a better discriminative ability

The DC in the right STG could be used to differentiate MDD from HC (AUC = 0.779). These results indicated that DC changes in the right STG can be considered an indicator of depression. The importance of DC in the STG has been emphasized because of its good discriminative ability. Similarly, the DC in the right MTG could be used to distinguish MDD from SD (AUC = 0.704). This indicates that changes in DC in the right MTG could be imaging biomarkers for distinguishing MDD from SD.

Logistic regression was used to regress the brain regions with significant differences in each comparison as one composite index and the three composite indexes had a good discriminative ability. This suggests that depression may not be caused by dysfunction or changes in a single brain region; instead, multiple brain regions and their interactions contribute to depressive symptoms. The diverse features can be used to take multiple aspects of the disease into account to estimate and classify MDD and SD. We can use machine learning models to conduct a comprehensive analysis of multiple features to obtain higher classification accuracy.

### 4.4. Limitations and future works

This work still has shortcomings. First, the accuracy of the statistical results was limited because the sample size was small. Second, we could not study the developmental trajectories of the same patients at different stages of depression because the multistage data for the same group of subjects was unavailable. In future work, data from the Strategic Research Program for Brain Sciences dataset (71) and the REST-meta-MDD Project (72) could be included. Furthermore, machine learning models can be used for more intelligent classification tasks of depression using DC or other graph theory features. After expanding sample sizes, deep learning models, such as graph neural networks (73), could also be used for classification.

## 5. Conclusion

This study analyzed the alterations in DC for patients with MDD and SD compared to HCs based on rs-fMRI data. Brain regions with altered DC values included the MFG, STG, MTG, and IPL. In the DC analysis, the right STG showed significant changes in the three pairwise comparisons. The alteration tendency for DC alteration of five important brain regions was identified. The DC of the right STG showed a good differentiation ability in the comparison between MDD patients and HCs, while the DC of the right MTG showed a good differentiation ability to distinguish MDD patients from SD patients. The three composite indexes showed good discriminative ability. These findings may help to explore patterns of functional brain networks in depressed people or identify biological markers of depression.

## Data availability statement

The datasets presented in this article are not readily available because the sharing of data must be approved by the Medical Ethics Committee. Requests to access the datasets should be directed to SQ, qisl@bmie.neu.edu.cn.

## Ethics statement

The studies involving human participants were reviewed and approved by Medical Ethics Committee of the affiliated Guangzhou First People’s Hospital of Guangzhou Medical University. The patients/participants provided their written informed consent to participate in this study.

## Author contributions

LY performed the experiments and analyzed the data along with CJ and SQ. SQ, YY, and XW conceived the study, presented the results, and wrote the manuscript along with LY. XR collected and analyzed the data. YT and CL supervised the algorithm development and analyzed the data. All authors read and approved the final manuscript.
